# Context-specific variation in life history traits and behavior of *Aedes aegypti* mosquitoes

**DOI:** 10.3389/finsc.2024.1426715

**Published:** 2024-09-25

**Authors:** Clément Vinauger, Karthikeyan Chandrasegaran

**Affiliations:** ^1^ Department of Biochemistry, Virginia Polytechnic Institute and State University, Blacksburg, VA, United States; ^2^ Department of Entomology, University of California Riverside, Riverside, CA, United States

**Keywords:** *Aedes aegypti*, context-specific variation, life history traits, behavioral plasticity, transstadial effects

## Abstract

*Aedes aegypti*, the vector for dengue, chikungunya, yellow fever, and Zika, poses a growing global epidemiological risk. Despite extensive research on *Ae. aegypti*’s life history traits and behavior, critical knowledge gaps persist, particularly in integrating these findings across varied experimental contexts. The plasticity of *Ae. aegypti*’s traits throughout its life cycle allows dynamic responses to environmental changes, yet understanding these variations within heterogeneous study designs remains challenging. A critical aspect often overlooked is the impact of using lab-adapted lines of *Ae. aegypti*, which may have evolved under laboratory conditions, potentially altering their life history traits and behavioral responses compared to wild populations. Therefore, incorporating field-derived populations in experimental designs is essential to capture the natural variability and adaptability of *Ae. aegypti*. The relationship between larval growing conditions and adult traits and behavior is significantly influenced by the specific context in which mosquitoes are studied. Laboratory conditions may not replicate the ecological complexities faced by wild populations, leading to discrepancies in observed traits and behavior. These discrepancies highlight the need for ecologically relevant experimental conditions, allowing mosquito traits and behavior to reflect field distributions. One effective approach is semi-field studies involving field-collected mosquitoes housed for fewer generations in the lab under ecologically relevant conditions. This growing trend provides researchers with the desired control over experimental conditions while maintaining the genetic diversity of field populations. By focusing on variations in life history traits and behavioral plasticity within these varied contexts, this review highlights the intricate relationship between larval growing conditions and adult traits and behavior. It underscores the significance of transstadial effects and the necessity of adopting study designs and reporting practices that acknowledge plasticity in adult traits and behavior, considering variations due to larval rearing conditions. Embracing such approaches paves the way for a comprehensive understanding of contextual variations in mosquito life history traits and behavior. This integrated perspective enables the synthesis of research findings across laboratory, semi-field, and field-based investigations, which is crucial for devising targeted intervention strategies tailored to specific ecological contexts to combat the health threat posed by this formidable disease vector effectively.

## 1 Introduction

The global epidemiological risk associated with *Aedes aegypti* is a significant concern. This invasive mosquito species is a crucial vector for several mosquito-borne viruses causing dengue, chikungunya, yellow fever, and Zika. These viruses are responsible for frequent outbreaks of diseases, leading to morbidity and mortality and substantial economic burdens worldwide. Dengue, in particular, has become a significant public health concern over the past decade, with approximately 3.9 billion people at risk of infection in over 128 countries (1, 2). Dengue incidence in the Americas, Southeast Asia, and the Western Pacific regions surged to approximately 5.5 million in 2020, as reported by the Pan American Health Organization (3). This worrisome upward trajectory has persisted, with 4.6 million dengue cases in the Americas alone in 2023, and over 9.3 million cases already reported as of June 2024 (4). In the United States, where cases were typically associated with international travel, the local transmission of dengue fever was reported in Arizona, California, Florida, Hawaii, and Texas in 2023, raising new challenges for local public health departments. Besides posing health risks, the cumulative economic costs of mitigating *Aedes*-borne diseases from 1975 to 2020 are estimated at 310.8 billion USD worldwide (5).

*Ae. aegypti* is the primary vector of dengue, chikungunya, Zika, and yellow fever viruses. *Ae. albopictus* also serves as a vector for these arboviruses, contributing to their transmission in various regions (6, 7). Since 1920, the estimated global abundance of *Ae. aegypti* has risen by approximately 9.5%, and future projections indicate a 30% increase by the end of the 21st century (8). By 2080, *Ae. aegypti* is predicted to be reported in as many as 162 countries, including countries documenting their presence for the first time (9).

Given these escalating risks, understanding the adaptability of *Ae. aegypti* to changing conditions becomes paramount for understanding its success as one of the most invasive mosquito species. Adaptability in *Ae. aegypti* encompasses genetic variation and phenotypic plasticity, each playing crucial roles in the mosquito’s ability to respond to environmental changes. Genetic variation provides the raw material for natural selection, enabling populations to evolve over time (10, 11). Influenced by larval environments, phenotypic plasticity allows individual mosquitoes to adjust their traits in response to immediate conditions. Moreover, plasticity can evolve within populations over time, potentially interacting with genetic differences. This means that the degree of genetic differentiation underlying various traits can vary within a population, leading to intrapopulation diversity in plastic responses that enhance adaptability (12, 13).

The plasticity of traits throughout their life cycle allows them to respond dynamically to environmental changes (14, 15). However, despite the acknowledged importance of adaptations and trait plasticity in *Ae. aegypti*, significant gaps persist in how we investigate and perceive behavior and life history trait variations, given the contextual complexity arising from heterogeneous study design and methodology. It is important to note that not all plastic responses are adaptive; some may arise from physiological or environmental constraints (16). Understanding genetic and plastic contributions to adaptability provides a comprehensive view of how *Ae. aegypti* can thrive in diverse environments.

While this review primarily focuses on *Ae. aegypti*, it is essential to consider insights from studies on other mosquito species to understand phenotypic variation and adaptability comprehensively. For instance, studies on *Ae. albopictus* and other mosquito species have highlighted similar adaptive responses to environmental pressures, suggesting broader patterns that can inform our understanding of *Ae. aegypti* (7). These comparisons can reveal fundamental principles of mosquito biology and adaptation, enhancing our ability to predict and manage vector populations.

This review highlights such knowledge gaps, specifically leveraging findings across laboratory, semi-field, and field-based investigations (17). By integrating insights from various *Aedes* species and other mosquitoes, this review emphasizes the importance of adopting study designs and reporting practices that acknowledge plasticity in adult behavior while also considering variation arising from differences in larval and adult traits due to larval growing conditions, also referred to as transstadial effects. Embracing such approaches paves the way for a comprehensive understanding of contextual variation in mosquito life history traits and behavior. This integrated perspective enables the synthesis of research findings across different study contexts, ultimately improving our capacity to devise targeted intervention strategies in the field tailored to specific ecological contexts to effectively combat the health threat posed by this formidable disease vector.

## 2 Variation in life-history traits

*Ae. aegypti* exhibits considerable variation in life history traits, contributing to its adaptability and vector potential. This review focuses on several key life history traits, including longevity, fecundity, adult body size, age of reproduction, and reproductive effort. These traits are influenced by both genetic and environmental factors, with phenotypic plasticity playing a crucial role in the mosquito’s adaptability. Understanding these variations is crucial because they provide insights into the mechanisms driving the adaptability and invasiveness of *Ae. aegypti*.

*Ae. aegypti* is a globally distributed and highly variable species, with significant variation in traits both within and among populations. Differences in genetic composition and environmental conditions across various geographical regions can lead to substantial differences in traits such as fecundity, longevity, development time, and vector competence (18, 19). This intra-species variability influences how different populations respond to environmental pressures and control measures, and it can affect disease transmission dynamics and invasion potential (20).

**Figure 1:**
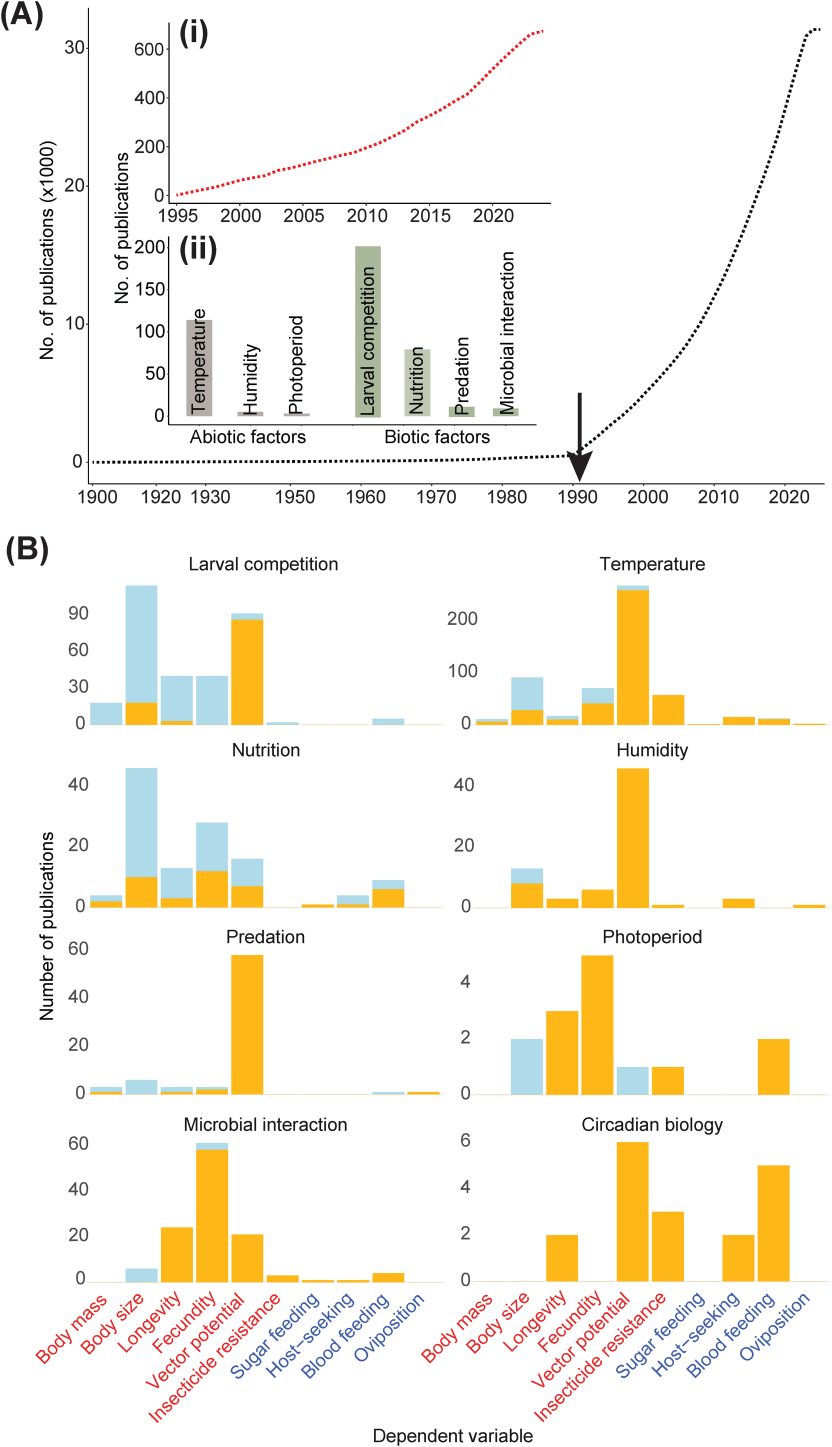
Trends in Mosquito Research Publications: **(A)** The cumulative number of publications from 1900 to 2024 focusing on life history traits and behavior of all mosquito species. The black arrow indicates the first description of the influence of transstadial effects. Insets: (i) Cumulative number of publications until 2024 specifically focused on *Ae. aegypti* traits and behavior. (ii) Cumulative number of publications investigating the impact of specific abiotic and biotic factors on *Ae. aegypti* life history and behavior. **(B)** A visualization of the literature trend investigating the effects of abiotic and biotic factors (independent variable) on larval and adult traits and behavior across all mosquito species. The bar plots in the left column represent the data on the number of studies investigating the effects of five biotic (larval competition, nutrition, predation, microbial interaction, circadian biology) and three abiotic factors (temperature, humidity, photoperiod) across all mosquito species. Along the X-axis are the most commonly investigated dependent variables; names denoted in red and blue denote adult traits and adult behavior, respectively. Yellow-shaded bars denote studies that have not considered the influence of transstadial effects on adult traits and behavior stemming from larval growing conditions. Blueshaded bars denote studies that have factored in transstadial effects. Data source: Clarivate Web of Science.

Mosquito life history traits and behavior have been well studied across multiple species (**Figure 1A**). Among these studies, population-specific variation in traits have been documented, showing that mosquitoes from different regions exhibit varying levels of insecticide resistance, which can impact the effectiveness of control strategies (21). Additionally, the genetic diversity within populations can influence their capacity to adapt to new environments, making some populations more successful invaders than others (22). Therefore, it is essential to consider genetic and environmental factors when studying the life history traits and vector potential of *Ae. aegypti*.

Several studies have documented adaptive plasticity in life-history traits, resistance to desiccation and insecticides, preference towards urban environments, and degree of anthropophily. However, the influence of environmental and physiological factors (i.e., context-specificity) in shaping the variations in these plastic traits (i.e., the direction and magnitude of effects) needs more attention. Many studies have investigated the influence of temperature on plasticity in life history traits, so we will discuss these findings considering the abundance of available data (**Figure 1B**). Additionally, we will discuss context-specificity in neuroethological studies because sensory processes involved with host detection and location have been extensively studied.

*Ae. aegypti* predominantly thrives in habitats with temperatures ranging from 18°C to 38°C, with the median temperature ranging between 25°C and 32°C (23, 24). While they are also found in much colder and warmer habitats, temperatures between 18°C and 38°C facilitate their complete metamorphosis, survival, and reproduction. These temperatures account for approximately two-thirds of their current geographical range (25, 26). Not surprisingly, the poleward shifts in their global distribution are predicted to covary strongly with mosquitoes’ adaptations to more extreme temperatures (26, 27).

The relationship between temperature and life history traits in *Ae. aegypti* is complex and often nonlinear. Many researchers view traits such as egg viability and larval survival as having optimal temperature ranges where the traits are maximized, with reduced viability and survival at temperature extremes (28, 29). For instance, egg viability typically peaks at intermediate temperatures and decreases at both lower and higher extremes (30). Similarly, larval survival rates tend to be highest within a moderate temperature range and drop off at temperatures outside this range, reflecting a nonlinear response (31). It is essential to recognize that temperature effects on most life history traits are better described by nonlinear or non-monotonic relationships, with trait performance often peaking at optimal temperatures and declining at suboptimal extremes.

While development rate is one of the few traits that might exhibit a more linear relationship with temperature within a limited range, even this relationship can become nonlinear at higher temperatures where development may fail due to mortality (32). The slope of this linear relationship corresponds to the cumulative effect of temperature variations on the development rate, and the intercept represents the theoretical temperature at which development ceases to occur, also known as the developmental zero (32, 33). However, empirical data from multiple studies suggests that this linear relationship is likely only true for mosquitoes developing within the median temperature range, i.e., 25°C and 32°C (34).

Outside the median temperature range, as temperatures approach the warmer or colder extremes, the magnitude of temperature-mediated effects scale non-linearly per unit change in temperature. For example, the egg hatch rates exhibited a non-linear decline with rising temperature, decreasing to 1.6% at 35°C. Similarly, lower temperatures also have had a non-linear impact on egg hatchability, albeit with a lesser magnitude of decline compared to higher temperatures: from 72% at 20°C to 55% at 18°C, 60% at 16°C, 53% at 14°C, and 43% at 12°C (30). Likewise, larval rearing at 27°C resulted in a pupation rate of 98.5% seven days post egg hatch, but this decreased to 97.2%, 87.4%, and 74.2% at 30°C, 33°C, and 35°C, respectively (35).

**Figure 2:**
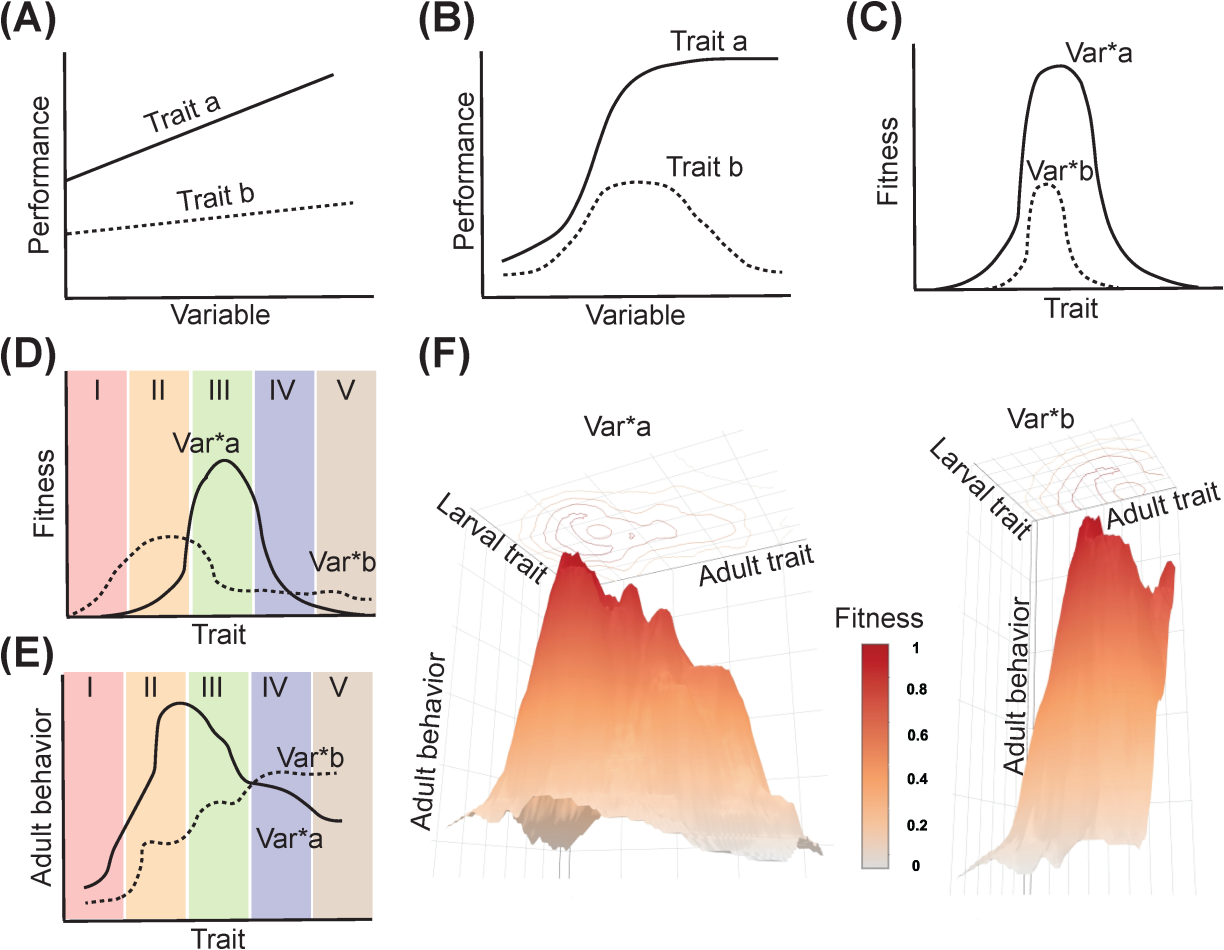
Adaptive trait-environment relationships in *Ae. aegypti*: **(A)** Classic representation of trait plasticity in response to environmental variables, **(B)** Representation of non-linear relationships between traits and environmental variables, **(C)** Influence of non-linear trait-environment relationships on mosquito fitness, **(D, E)** Existing methods fail to consider the covariation in larval and adult traits and its impact on adult behavior and fitness within the framework of environment-trait relationships. Sections I-V illustrate hypothetical segments of the overall trait distribution and demonstrate how sampling only a subset of this distribution affects the interpretation of relationships between fitness, adult behavior, and life-history traits, **(F)** A comprehensive framework that visualizes the data in **(D, E)** by depicting the environment-trait-fitness relationship while accounting for the covariations in larval and adult traits influenced by transstadial effects. This visualization assumes a 1:1 correlation between environment-trait variables and fitness; therefore, the color gradient mirrors the 3D surface. However, this correlation may vary across specific experiments. Asterik (*) symbol in **(C–F)** denote the interaction between two variables.

Even within the median temperature range, the relationship is often nonlinear when the effects of temperature have been studied in interaction with other environmental factors. For instance, larval competition and resource availability affect the temperature dependence of *Ae. albopictus*’s fitness (36). In particular, in resource-scarce or high-competition environments, the temperature facilitating optimal development and fitness drops by ~6°C. Furthermore, these interactive effects result in a ~10°C reduction in the width of *Ae. aegypti*’s thermal niche, *i.e.*, the range of temperatures that facilitates the species’ survival and reproduction (37). To better visualize this context, **Figures 2A–C** presents a hypothetical illustration highlighting the differences between modeling the environment-trait-fitness relationship as linear versus nonlinear. **Figure 2A** depicts a classical linear environment-trait relationship. **Figure 2B** illustrates the nonlinear relationship, as discussed in the examples above on temperature-mediated effects on life history traits. **Figure 2C** shows how these environmentally-mediated trait variations shape mosquito fitness.

In addition, the interactions between temperature and relative humidity, together with variation in adult body size, strongly correlate with the longevity of adult *Ae. aegypti* mosquitoes (38). Low resource larval environments at 26.4°C resulted in females with shorter lifespans (6.9 days) compared to larval environments with similar resource availability at 30.1°C (10.7 days) and 35.1°C (8.5 days) (38). Similarly, the time taken to reach pupation decreased progressively, exhibiting a non-linear decline, with durations of 21.97 days at 15.2°C, 14.46 days at 17.9°C, 9.83 days at 21.6°C, and 8.67 days at 25.3°C (39). These non-linear temperature-mediated effects highlight the complexity of *Ae. aegypti*’s response to temperature fluctuations and suggest potential analogous trends in other life-history responses to various biotic and abiotic factors.

In the context of reproduction, higher temperatures lead to reduced egg production in *Ae. aegypti* females, reduced the latency to oviposition, and altered oviposition patterns. For instance, at 25°C and 80% humidity, *Ae. aegypti* females lived twice as long and produced 40% more eggs than at 35°C and 80% humidity. At high temperatures and high humidity, mosquitoes survived less and produced fewer eggs. At 35°C and 60% humidity, only 15% of females laid more than 100 eggs, and 45% of the females did not oviposit any eggs. Egg fertility also decreased with rising temperatures at lower humidity levels (40). The interaction between temperature and humidity plays a crucial role in the survival of eggs. High humidity levels enhance egg viability and hatching rates at optimal temperatures, while low humidity leads to desiccation and reduced viability, even if the temperature is within a favorable range (41). This interaction highlights the complexity of environmental factors affecting mosquito life history traits. Moreover, the magnitude of any larval environmental effect on adult traits differs between male and female mosquitoes due to protandry, whereby female mosquitoes exhibit a slower growth rate than their male counterparts (42, 43). Consequently, females spend more time in larval habitats, rendering the quality of larval growing conditions significantly more influential on female larval and adult traits than males. For example, suboptimal larval growing conditions generally lead to higher female mortality rates and a skewed sex ratio towards males (43). Density-dependent female larval mortality is also a critical determinant of adult body size and other traits (44). Thus, evaluating plasticity in mosquito traits requires a sex-specific approach.

Understanding the distribution of life history traits is essential for comprehending the biological and ecological factors contributing to *Ae. aegypti*’s success as an invasive species and a vector. Longevity and fecundity, for example, are directly related to the mosquito’s ability to sustain and spread infections over time (45). Variation in the age of reproduction can lead to differences in generation time, affecting how quickly populations can grow and adapt to new environments (46). Adult body size is another critical factor influencing mosquitoes’ survival, fecundity, and vector competence (23). Larger adult mosquitoes generally have higher fecundity and longer lifespans, making them formidable disease vectors (1). However, body size is highly influenced by larval rearing conditions, such as temperature, food availability, and density. For example, larval competition and limited resources can lead to smaller adult sizes, which may reduce individual survival and reproductive success (47). Notably, these factors can result in similar phenotypic outcomes through different mechanisms. The reproductive effort reflects the balance between the number of offspring produced and the investment in each offspring’s quality, significantly impacting population dynamics and resilience (1). These life history traits are influenced by both genetic and environmental factors, with phenotypic plasticity playing a crucial role in how *Ae. aegypti* adapts to varying conditions (14, 43). Therefore, deciphering the interplay between these environmental factors and life history traits enhances our comprehension of the complex biology of *Ae. aegypti* and sheds light on the adaptive mechanisms that make them such formidable disease vectors.

## 3 Genetic variation and phenotypic plasticity

While phenotypic plasticity plays a crucial role in the adaptability of *Ae. aegypti*, it is essential to recognize the genetic components underlying these traits. Phenotypic traits such as host choice, behavior, and adult size exhibit significant genetic variation, which interacts with environmental factors to shape the observed phenotypic outcomes (48, 49). This interaction between genetic variation and phenotypic plasticity is pivotal for understanding the adaptability and vector potential of *Ae. aegypti* (15, 50).

Host choice exemplifies a trait influenced by both genetic and environmental factors. Studies have shown that *Ae. aegypti*’s preference for human hosts has a strong genetic basis, with certain populations exhibiting innate tendencies towards anthropophily (51). Specific genetic loci associated with the preference for human odors underscore the genetic underpinnings of this behavior. However, environmental conditions, such as the availability of hosts and habitat characteristics, also modulate this preference, showcasing epigenetic plasticity.

Behavioral traits, including feeding and oviposition behaviors, also exhibit genetic variation. For instance, the genetic differentiation between sylvatic and domestic forms of *Ae. aegypti* influences their behavior and habitat preferences (52). Sylvatic populations tend to feed on a broader range of hosts and oviposit in natural habitats, while domestic populations strongly prefer human hosts and artificial containers for oviposition. These inherent genetic differences are further influenced by environmental factors, such as the availability of breeding sites and host density, leading to context-specific behavioral adaptations.

Adult size is another trait where genetic variation and phenotypic plasticity intersect. Body size is determined by both genetic factors and larval rearing conditions, such as temperature and resource availability (53). Genetic differences between populations can result in varying growth rates and adult size at emergence (15). Heritability plays a significant role in determining adult body size, yet environmental factors like larval density and nutrition levels also induce plastic responses, affecting size-related traits such as longevity and fecundity. This interaction between genetic predisposition and environmental conditions underscores the complexity of size variation and its implications for vector competence (15).

Understanding the interplay between genetic variation and phenotypic plasticity is crucial for predicting how *Ae. aegypti* populations might respond to changing environments (49). This knowledge is vital for devising effective vector control strategies, as both genetic adaptation and plastic responses can influence the success of interventions. For example, genetic differences in insecticide resistance can interact with environmental factors, such as exposure levels and habitat characteristics, to shape resistance dynamics (48). Recognizing the contributions of both genetic variation and phenotypic plasticity will enhance our ability to anticipate and manage the evolutionary responses of *Ae. aegypti* to control measures.

## 4 Behavioral plasticity

The success of *Ae. aegypti* as an invasive species is largely attributed to its anthropophilic behavior, with a strong preference towards human hosts for blood meals. While its host-seeking behavior has been extensively studied, its sugar-feeding habits and preferences, crucial for metabolic sustenance, remain relatively unexplored (54). Given the exclusive sugar-feeding diet of males and the importance of carbohydrates for females’ metabolism, this is a crucial contributor to the species’ invasion potential (55). Despite *Ae. aegypti* mosquitoes predominantly inhabiting human-dominated areas, it is essential to acknowledge the existence of known sylvatic populations that defy this trend and also feed on non-human hosts (52).

The variation between sylvatic and domestic forms of *Ae. aegypti* is a key example of both genetic differentiation and behavioral plasticity. Sylvatic *Ae. aegypti* primarily inhabit forested areas and utilize natural water containers for breeding, while domestic *Ae. aegypti* thrive in urban environments, breeding in artificial containers and frequently entering human dwellings. Multiple studies provide substantial evidence for a genetic basis underlying the plasticity in mosquito behavior (51, 56–58). These studies demonstrate that genetic differences are responsible for variations in behaviors such as host preference, habitat selection, and oviposition site choice. In addition to genetic differentiation, both *Ae. aegypti* and *Ae. albopictus* exhibit considerable flexibility in their feeding behaviors, which further reflects their adaptability to varying environmental conditions. For instance, while longevity typically decreases in the absence of sugar, fecundity can actually increase when females rely solely on human blood without sugar supplementation (59). This plasticity in feeding and reproductive strategies underscores the adaptability of these species, allowing them to thrive under different environmental pressures. Such flexibility is a testament to the complex interplay between diet, genetic predispositions, and life history traits that enable these mosquitoes to exploit a wide range of ecological niches. This combination of genetic factors and behavioral plasticity is central to the success of *Ae. aegypti* in invading tropical and subtropical regions. The inherent genetic differences between sylvatic and domestic forms are a result of their adaptation to specific environments, while behavioral plasticity enables them to respond dynamically to varying habitat conditions.

To identify and locate their hosts, *Ae. aegypti* females rely on the synergistic integration of sensory cues, including olfactory, visual, thermal, and gustatory cues (60). Olfactory cues and carbon dioxide facilitate long-range attraction, and other sensory cues enable medium-to-short-range attraction. Finally, thermal cues are primarily effective at short distances since temperature gradients created by convection around a human host diminish quickly (61–63). While there is a consensus on the relative significance of these sensory cues, the contribution of thermal cues in host-seeking behaviors is still debated, most likely due to contextual differences in the experimental paradigms used in studies. For example, some studies found that the attraction of *Ae. aegypti* to thermal cues depend on their ability to sense CO_2_ (64), while others showed that their attraction to thermal cues could also occur independently of CO_2_ (61). In the absence of CO_2_, the sensory integration of thermal and chemosensory cues (host volatiles) likely drives the host-seeking behavior (65). Nevertheless, there is evidence that thermal cues alone (specifically convective but not radiative heat) are sufficient for females to locate heat sources in the range of potential hosts’ temperature (66).

In the context of long-range attraction, the response to CO_2_ predominantly drives host-seeking and reduces the response threshold to human-derived odors. *Ae. aegypti*’s preferences for visual cues of specific wavelengths are also gated by the detection of CO_2_ (60). However, within a short range to the human host, orientation and landing behaviors are mediated by olfactory cues, not CO_2_ or a co-located visual cue (67, 68). Further, several hundred human-derived chemoattractants have been identified (69, 70). The valence of these chemoattractants can vary significantly, both in isolation and in synergistic combinations, and these variations are influenced by physiological and environmental factors (51, 71, 72).

While host-seeking is typically regarded as a female mosquito trait, *Ae. aegypti* males also display attraction to humans (73, 74). Mature males are particularly drawn to human-derived chemosensory cues, which drive their swarming behavior in pursuit of potential mates and contribute to their mating success in natural populations (66, 75). Amidst debates on mosquitoes’ attraction to sensory cues and multimodal sensory integration, responses to environmental cues in *Ae. aegypti* are often interpreted as phenotypic plasticity, where organisms modify their behavior or traits in response to changing environmental conditions (13). For instance, variations in host-seeking behavior can be influenced by larval rearing conditions, indicating plastic responses to environmental cues (23). However, some responses are considered innate, meaning they are hardwired into the organism’s genetic makeup and are not easily altered by the environment (51). These innate responses can still exhibit context-specific variations, as the expression of innate traits can be modulated by environmental factors (76). Thus, distinguishing between plastic and innate responses requires careful consideration of both genetic and environmental influences (77). Unfortunately, many studies overlook this context specificity, focusing on mechanistic effects under controlled conditions. While mechanistic insights are valuable, the broader relevance of these mechanisms in real-world contexts is needed to bridge findings from the lab to their applicability in field conditions.

Extrinsically, factors like temperature, humidity, and photoperiod significantly shape mosquito behavior. As poikilotherms, the ambient environmental temperature dictates mosquitoes’ body temperature and activity. Warmer temperatures, for example, increase their metabolism, leading to heightened activity and increased sugar feeding from plants to meet their nutritional needs (78). Besides elevated activity levels, temperature influences the sensitivity to chemosensory cues as *Ae. aegypti* females were more attracted to CO_2_ when tested at 30°C compared to 20°C and 25°C (79). Electrophysiological recordings indicate that odorant-specific changes in antennal sensitivity to odors mediate this effect of temperature on olfactory behavior (79). The differences in response to odorants could also be partly due to temperature-induced alterations in the characteristics of odorant compounds. Indeed, one can expect temperature changes to affect the chemicals’ partial vapor pressure, impacting their diffusion and subsequent interaction with odorant binding receptors in the chemosensory organs of *Ae. aegypti* (80, 81).

Intrinsically, as observed in many other insect models, the olfactory sensitivity of *Ae. aegypti* varies with the time of day (82), with several genes involved in olfactory processes being regulated by the mosquito’s circadian clock (83, 84). Furthermore, sleep deprivation detrimentally affects both host-seeking and blood-feeding behaviors in *Ae. aegypti*, potentially linked to alterations in time-dependent olfactory sensitivity (85). It is thus critical to synchronize mosquitoes to test behavioral and physiological responses to host cues in the proper (or most relevant) temporal context. It is also essential to report temporal information in publications as the norm. Overall, the physiological state of the insect (*e.g.*, age, reproductive status, feeding state, chronobiology, sleep patterns, prior experience, *etc.*) significantly affects its responses to resource-associated cues (86). For example, older females, who typically exhibit a higher propensity for seeking hosts, display increased sensitivity to CO_2_ compared to their younger counterparts in the first few days post-emergence. (87). Additionally, mating and blood-feeding suppress host-seeking behavior, with a return to baseline levels after oviposition (88–90).

Altogether, this underscores the importance of considering the interplay between mosquitoes’ physiology and behavior. Overall, extrinsic and intrinsic factors modulate mechanisms at peripheral and central levels to drive behavioral variation tailored to different physiological and ecological contexts.

## 5 Using transstadial effects to navigate contextual complexity in studying life history and behavior

The variation in life history traits and behavior of *Ae. aegypti* described in the literature are primarily influenced by decisions made by experimenters, *i.e.*, the study design employed to quantify traits. While simple experimental designs offer more explicit contexts, their capacity to fully capture the breadth of variation in mosquito life history and behavior is debatable. On the other hand, complex study designs reporting multivariate and interactive effects encounter challenges in discerning the relative contribution of each independent variable to the magnitude and direction of observed effects on traits and their underlying distribution (91). These studies also hinder experimenters’ ability to dissect the mechanistic underpinnings of the observed behaviors (49). This complexity arises when multiple experimental variables are manipulated simultaneously, potentially interacting in ways that obscure context-dependent effects unless addressed explicitly in the study design (92–94).

Numerous studies have explored the impact of larval competition and nutrition on adult mosquito body size at emergence. Typically, heightened larval competition and lower nutrition reduce per capita resource availability, resulting in smaller adult mosquitoes (95, 96). Consequently, a diverse range of mosquito size distributions resulting from varying levels of larval competition and nutrition have been reported in the literature (43, 97–99). However, it remains challenging to determine if these varying size distributions across studies, often overlapping, genuinely reflect the ecologically relevant limits (*i.e.*, in larval density and food availability). To address this concern, some studies integrate field-derived preliminary data on larval density or food availability in natural habitats, ensuring that experimental variables are manipulated within ecologically relevant bounds (100, 101). Others employ experimental variables that may not strictly adhere to these limits but conduct standardization trials under controlled conditions to establish the range within which variables can adequately capture a trait’s distribution (102, 103). When manipulated individually, varying larval densities and food levels could still produce adults with similar size distributions, although due to different physiological responses. Nevertheless, more needs to be understood about whether these adults, despite their similar size distribution, share similarities in their physiological, behavioral, and life history characteristics. When these variables interact, untangling their influence on adult size distribution becomes complex and challenging. Nonetheless, these intricacies are frequently disregarded in many studies, highlighting the significance of interpreting effect sizes on mosquito traits and behavior while considering the distribution’s shape, especially at the tails, where sampling efforts may be limited. These gaps in approach will likely impact the perceived relationship between environmental factors and life history traits, the extent of variation, and the plasticity window for those traits (**Figures 2D, E**).

It is important to consider whether the source of size variation, or any other phenotypic variation, influences how these traits relate to fitness and disease transmission. Different sources of variation, whether genetic, physiological, or environmental, might have distinct effects on these outcomes. Investigating these differences could provide valuable insights into the adaptive strategies of *Ae. aegypti*. This question merits further exploration in the context of understanding vector competence and developing effective control strategies. For example, resource limitation and competition during larval development often result in smaller adults, which can exhibit greater susceptibility to several arboviruses and, in some cases, higher oral transmission rates (47, 104). Conversely, other studies have found that smaller individuals from high-density rearing conditions may have reduced vector competence (105, 106). Additionally, research on *Ae. albopictus* has indicated that higher temperatures during larval development can produce smaller adults with lower susceptibility to some arboviruses.

Transstadial effects, such as resource limitation, fluctuations in temperature and humidity, predator presence, parasites and several other factors, during larval development, significantly influence the adult size of mosquitoes, primarily through phenotypic plasticity. As previously noted, these factors may lead to similar phenotypic outcomes through different mechanisms. However, the role of selection among individuals with inherent genetic differences in size in shaping adult size distributions under various larval growing conditions has been less frequently considered. Additionally, size distributions can vary among populations due to inherent genetic differences (107). Future research investigating the specific contributions of genetic and environmental factors to mosquito size variation, mediated by transstadial effects, is crucial for understanding their impact on fitness traits and vector competence in *Ae. aegypti* and other mosquitoes.

Multiple studies have revealed intriguing trends linking variation in temperature with the host-seeking behavior of female *Ae. aegypti*. However, a crucial aspect is often overlooked: mosquitoes’ complex life history and the influence of larval growing conditions that cascade to modulate adult traits and behavior (43, 76, 108, 109). Temperature-driven variation in host-seeking behavior have often been reported during behavioral studies (110, 111). The complex life cycle of *Aedes* mosquitoes involves distinct habitats for larvae and pupae versus adults, which often results in them experiencing different thermal environments. Larvae and pupae are confined to aquatic environments, typically in small volumes of water (single containers, tree holes, etc.), with a narrow thermal range during their development. In contrast, adult females are highly mobile and traverse various aerial and terrestrial environments, exposing them to a wide range of microclimatic conditions as they seek sugar sources, hosts for blood meal, oviposition, and resting sites (112, 113).

Given these differing thermal exposures, it is essential to consider how transstadial effects might influence sensory and behavioral responses. The thermal mismatch or match between larval and adult environments could significantly impact adult mosquitoes’ phenotypic traits and behaviors. For example, a stable larval thermal environment might lead to different developmental outcomes compared to the variable thermal exposures experienced by adults, affecting their survival, reproduction, and vector competence (28 and 114–116). Studies have shown that discrepancies in thermal environments between life stages can alter adult behavior and physiology, emphasizing the need for experimental designs to account for these differences (114). Therefore, the thermal history of both larval and adult stages is crucial for accurately interpreting the effects of temperature on mosquito biology and behavior. This consideration helps ensure that experimental results are ecologically relevant and reflective of the natural conditions experienced by mosquitoes throughout their life cycle. However, the adult mosquitoes used in most behavioral studies often originate from larval environments chosen primarily for optimizing the yield of colonies, needing more alignment with the experimental context. (64, 117). Also, it is essential to note that most behavioral studies need to accurately report the larval growing conditions, which hinders contextualizing the reported effect sizes on adult behavior.

Laboratory studies often focus on controlled conditions to ensure repeatability and reliability of results. However, these studies might not fully capture the complexity and variability of mosquito behavior in the wild. For instance, despite artificial blood feeding over many generations, laboratory strains of *Ae. aegypti* maintain a strong preference for human hosts, demonstrating that some behaviors are robust and persist even under artificial rearing conditions (19, 56, 118). Nevertheless, many other behaviors and responses might be context-specific and influenced by the natural environment (*e.g.*, circadian rhythms, activity patterns, etc.), which laboratory settings fail to replicate completely (119). Therefore, a hypothetical framework is needed to interpret the environment-trait-fitness relationship, considering the covariations in larval and adult traits influenced by transstadial effects (**Figure 2F**). This framework emphasizes the importance of visualizing the covariations in larval and adult traits, considering the influences of independent variables across developmental stages along the two axes. Such an approach is crucial for accurately interpreting their effects on adult behavior (third axis) and its implications for mosquito fitness (fourth dimension). By accounting for these relationships between environmental variables, transtadially-mediated trait variation and adult fitness, we can better contextualize experimental findings and highlight their ecological relevance.

Furthermore, adult females selected for behavioral assays from laboratory colonies may exhibit trait distributions specific to their rearing conditions, such as larval crowding, feeding regime, temperature, and humidity (120–124). As described in several studies’ methodology, the typical “standard larval rearing condition” often does not yield adult *Aedes mosquitoes* representing the full spectrum of their trait distributions for use as subjects in behavioral experiments (28, 32, 125–127). Critically, this limitation extends beyond laboratory-reared mosquitoes to field-collected mosquitoes utilized in laboratory and semi-field experiments (128, 129). In such instances, the comprehensive trait distribution is frequently disregarded, resulting in the unintentional selection of mosquitoes with a subset of trait values or characteristics as experimental subjects. Due to the strong covariation between life-history traits and several adult behaviors, studies conducted with these mosquitoes, representing only a subset of the overall trait distribution, may only incompletely capture the variation associated with a specific behavioral repertoire. This limitation not only narrows the range of contexts in which study results can be interpreted but also affects the magnitude and direction of reported effect sizes, presenting challenges for reproducibility and generalization to broader contexts.

## 6 Discussion

From a vector-borne disease control perspective, studies on mosquito life history traits and behavior aim to elucidate their significance in influencing vector potential, insecticide resistance, and invasion potential. This review highlights how neglecting context-specific effects significantly undermines the accuracy of the relationships quantified between experimental variables. By integrating findings from studies on other species, we can draw broader conclusions and identify patterns that may apply across mosquitoes. For example, similar phenotypic plasticity and adaptability mechanisms observed in *Ae. albopictus*, another important vector species, can provide comparative insights that enhance our understanding of *Ae. aegypti*. Such cross-species comparisons are particularly valuable for identifying generalizable principles of mosquito biology and vector management, which can inform strategies to control multiple species simultaneously.

Moreover, considering genetic and phenotypic variation across mosquito species helps us recognize the evolutionary pressures and environmental factors shaping these traits. This understanding is crucial for anticipating how mosquito populations may respond to environmental changes, such as climate variability, urbanization, and habitat modification. For instance, the genetic diversity within populations can influence their capacity to develop insecticide resistance, necessitating the development of dynamic and adaptable control measures. Additionally, recognizing the role of phenotypic plasticity in facilitating rapid adaptation to new environments can help predict and mitigate the spread of invasive mosquito species.

The lack of context specificity in reported effects, along with difficulties in experimentally quantifying population-specific variation in mosquito traits and behavior, complicates the parameterization of data for predicting mosquito demography, distribution, and disease transmission dynamics (44, 49, 130–132). For instance, the impact of larval rearing conditions on adult traits is often studied using laboratory-bred strains under controlled laboratory conditions, which may not accurately reflect the variability encountered in natural settings. This discrepancy can lead to over- or underestimation of the effects of environmental factors on mosquito populations.

Unfortunately, interactive effects are frequently viewed as epistemically precarious due to their variability, sometimes leading to the belief that observed effect sizes are unpredictable (133–136). This perspective stems from the challenge of isolating specific variables in multifactorial experiments and the inherent complexity of ecological interactions. However, this review stresses that while interactive effects vary significantly across contexts, their variability does not imply unpredictability. With appropriate study design measures established *a priori*, or at least detailed, *a posteriori* reporting of experimental methods, their variability across contexts can be systematically studied, allowing knowledge to be rigorously extrapolated. Incorporating field-derived data into laboratory experiments is one such approach, enabling researchers to better simulate natural conditions and account for context-specific effects.

There are, however, notable exceptions to this issue. Some studies have successfully accounted for interactive effects, offering valuable insights into mosquito life-history traits. For example, Carrington et al. (2013) critically analyzed the effects of fluctuating daily temperatures on *Ae. aegypti*, emphasizing the interaction between mean temperatures and temperature fluctuations. Similarly, Muturi et al. (137) examined the interactive effects of temperature and insecticide exposure on the life-history traits of *Culex restuans* and *Ae. albopictus*, providing a nuanced understanding of how these factors together influence development time, survival, and adult size. Additional studies by Alto and Juliano (138) on temperature and larval density and Yeap et al. (139) on temperature and *Wolbachia* infection further illustrate how these environmental factors jointly shape mosquito traits and vector competence. These examples highlight the importance of considering interactive effects in mosquito research to avoid misinterpretation of biological outcomes and to ensure the ecological validity of laboratory findings.

By addressing these interactive effects in mosquito research, we can fully leverage the extensive knowledge gained from laboratory and semi-field studies, which outnumber field-based studies, to apply these findings effectively in field contexts. This integration is essential for developing accurate mosquito behavior and population dynamics models, which are critical for predicting or, eventually, mitigating vector populations and vector-borne diseases. Furthermore, understanding the interaction between genetic variation and phenotypic plasticity can help identify potential targets for genetic modification or biological control strategies, offering new avenues for disease prevention.

Overall, this review underscores the necessity of a holistic approach incorporating genetic, phenotypic, and environmental factors to understand mosquito ecology comprehensively. Such an approach will enhance our ability to develop robust, context-sensitive interventions that can adapt to the dynamic nature of mosquito populations and the environments they inhabit.
